# Economic evaluations in water-fluoridation: a scoping review

**DOI:** 10.1186/s12903-020-01100-y

**Published:** 2020-04-16

**Authors:** Rodrigo Mariño, Carlos Zaror

**Affiliations:** 1 Melbourne Dental School, University of Melbourne, Melbourne, Australia; 2 Department of Pediatric Dentistry and Orthodontics; Faculty of Dentistry, Universidad de La Frontera, Manuel Montt #112, Temuco, Chile; 3 Center for Research in Epidemiology, Economics and Oral Public Health (CIEESPO), Faculty of Dentistry, Universidad de La Frontera, Temuco, Chile

**Keywords:** Fluoridation, Cost-benefit analysis, Health economics, Oral health

## Abstract

**Background:**

Community water fluoridation (CWF) is considered one of the 10 greatest public health achievements of the twentieth century and has been a cornerstone strategies for the prevention and control of dental caries in many countries. However, for decision-makers the effectiveness and safety of any given intervention is not always sufficient to decide on the best option. Economic evaluations (EE) provide key information that managers weigh, alongside other evidence. This study reviews the relevant literature on EE in CWF.

**Methods:**

A systematic database search up to August 2019 was carried out using MEDLINE, EMBASE, Cochrane Library, LILACS, Paediatric Economic Database Evaluation and National Health Service Economic Evaluation Database. The review included full economic evaluations on CWF programs, written in English, Spanish or Portuguese. The selection process and data extraction were carried out by two researchers independently. A qualitative synthesis of the results was performed.

**Results:**

Of 498 identified articles, 24 studies met the inclusion criteria; 11 corresponded to cost-benefit analysis; nine were cost-effectiveness analyses; and four cost-utility studies. Two cost-utility studies used Disability-Adjusted Life Years,, one used Quality-Adjusted Tooth Years, and another Quality-Adjusted Life Years. EEs were conducted in eight countries. All studies concluded that water fluoridation was a cost-effective strategy when it was compared with non-fluoridated communities, independently of the perspective, time horizon or discount rate applied. Four studies adopted a lifetime time horizon. The outcome measures included caries averted (*n* = 14) and savings cost of dental treatment (*n* = 4). Most of the studies reported a caries reduction effects between 25 and 40%.

**Conclusion:**

Findings indicated that CWF represents an appropriate use of communities’ resources, using a range of economic evaluation methods and in different locations. These findings provide evidence to decision-makers which they could use as an aid to deciding on resource allocation.

## Introduction

Dental caries is the most prevalent chronic disease in the world today, and affecting a significant proportion of the world’s population [1]. In permanent dentition, dental caries is the most prevalent condition, affecting 34.1% of the world’s population (2.5 billion people) according to the global morbidity burden study of 2015 [1]. In primary dentition, dental caries is the 10th most prevalent condition, affecting 7.8% of the population, that is, 573 million children worldwide [1]. The health consequences of dental caries are serious and this disease can also greatly affect the quality of life of those who suffer from it [2].

While factors including the consumption of refined carbohydrates were responsible for the rise of caries in the first half of the last century, exposure to fluoride has had a crucial role in improving oral health during the past few decades [3]. Fluoride is the leading strategy in the noninvasive management of dental caries, and it has been incorporated as a public health measure to reduce the prevalence of that disease [4]. Fluoride’s main mechanism is topical, stimulating the remineralization of early carious lesions and reducing the risk of demineralizing healthy enamel [4]. Fluoride is now accepted as a safe, effective, efficient and appropriate mechanism for the prevention of dental caries [5]. Indeed, the use of fluorides is recognized as one of the most successful measures for the prevention of disease in the history of public health [6]. Fluoride can be delivered to individuals as a dental preventive measure through a variety of mechanisms. It can be administered systemically through water, milk, or salt or topically available as toothpastes and other dental products such as varnishes, gels and mouthwashes [5].

In the mid-1940s, fluoride was first adjusted to community water fluoridation (CWF), it has since then been introduced to the public drinking water in 26 countries around the world. The adjustment of fluoride content to optimal levels (0.6–1.0 ppm or 0.6–1 mg/L) in community water is the basis of prevention and control strategies for dental caries in those countries [5]. Currently, fluoride adjusted CWF benefits 372 million people. In addition, another 57 million people in 27 countries receive this benefit from water supplies that are naturally fluoridated [7]. The evidence indicates that a 26 to a 35% reduction in dental caries can be achieved with this public health measure in permanent and primary dentition, respectively [8].

The effectiveness and safety of any given intervention may not be enough to decide on its implementation. Other factors, including cost-effectiveness, as well as the political, organizational, social, ethical and legal impacts must be considered, especially when applied within the public health context [9]. Health service managers, programmers and planners are required to select the interventions with the highest impact, however these interventions come at a cost so planners need to assess the relative efficiency of interventions. Economic evaluations (EE) describe the relative effectiveness of interventions in comparison to the relative costs, this approach enables planners to identify those interventions that can maximize health for a given budget. It is now well established and recognized that EE is a central component of the objective evaluation of new technology/therapies and preventive programs that seek to replace current treatments or practices [10].

An economic evaluation is defined as “the comparative analysis of alternative actions in terms of their costs and their consequences in order to assist in policy decision” [11]. There are several important components to this definition, the first is that an economic evaluation must compare alternative interventions. Secondly, an economic evaluation measures not only costs, but also results or consequences. Thirdly, the technique represents only one dimension within a broader, integrated and cyclical decision-making and evaluation process [11]. Table 1 summarizes the economic evaluation process. Full details of economic evaluation methods, as applied to healthcare, can be found elsewhere [11].

**Table 1:** Steps for economic evaluation

**Step 1.** Define objective of the economic evaluation	
**Step 2.** Define economic evaluation framework	
• Perspective of the economic analysis	
• Alternatives being compared	
• Time horizon	
**Step 3.** Determine costs and benefits of alternatives	
• Define all activities	
• Specify measurements	
• Collect cost data	
• Calculate costs	
• Discount	
• Define outcomes	
• Select evaluation design	
• Collect data	
• Analyze data	
**Step 4.** Relate costs to outcomes	
• Ratio	
**Step 5.** Adjust for uncertainties	
• Sensitivity analysis	
**Step 6.** Summarize, Interpret, and report findings	

Modified from: Splett [12]

In the context of diminishing public resources for oral health care and increasingly sophisticated treatment options, decision-makers may not have enough information to identify the financial benefit per monetary unit of resources required for most health interventions. The need to understand health and healthcare systems and how to best allocate scarce resources, requires decision-makers to apply the full range of methods and skills to assure these resources are used wisely [13]. It is in this context that economic evaluations (EE) are relevant, as they provide information that managers weigh, alongside other evidence. Nonetheless, although, there are examples in the literature of EE in oral health, until recently, its overall use in oral health was limited. Recent reviews indicate that this is changing [14].

As the importance of economic evaluation in oral health will increase in the future, to make this process relevant to policy makers, this manuscript will review the relevant literature on EEs in CWF, describing their characteristics and reviewing their effectiveness and limitations.

## Methods

### Sources of information and search strategy

This scoping review was reported according to the Preferred Reporting Items for Systematic reviews and Meta-Analyses extension for Scoping Reviews (PRISMA-ScR) guidelines [15]. A systematic search of the literature published up to August 15, 2019, was conducted to identify existing economic evaluations of water-fluoridation. The following databases were used: MEDLINE (via PubMed), EMBASE, Cochrane Library, LILACS, Paediatric Economic Database Evaluation (PEDE), National Health Service Economic Evaluation Database (NHS EED). Because some economic evaluations about water fluoridation are not published in the academic literature, reference listings of retrieved articles and previous systematic reviews were hand searched to identify other possible studies. The details of the search strategy are given in an additional file.

### Study selection

Full EEs comparing both the costs and consequences of water fluoridation with status-quo or other interventions [11], in English, Spanish and Portuguese were included. Excluded were discussion papers, reviews and study protocols and incomplete economic evaluation, that is those where only the costs or consequences of water fluoridation were examined.

All references identified were extracted to an EndNote X9 database to facilitate their management and duplicate articles were eliminated. Articles were selected by title and abstract, and then by full text according to the eligibility criteria, using Rayyan online software (https://rayyan.qcri.org), with two researchers (RM and CZ) working independently. If there was a discrepancy, a consensus was reached. The reviewers were not blinded to the authors or journals. The reasons for exclusions were recorded.

### Data extraction

Two reviewers independently extracted data from each article that met the inclusion criteria, through a standardized spreadsheet. No quantitative analyses were performed beyond descriptive statistics to summarize findings. Rather a qualitative synthesis was performed. Extracted information included: author, year, country, type of economic evaluation, perspective, source of effectiveness outcome measure, outcome measure, time horizon, discount rate, price year, currency unit and main results.

Eligible studies were critically appraised by two independent reviewers (CZ and RM) at the study level for methodological quality using the Joanna Briggs Institute (JBI) Checklist for Economic Evaluations [16]. The level of methodological quality was determined as follows: fair quality = less than 40% of the items presented; moderate quality = between 41 and 80% of the items presented; good quality = more than 80% of the items presented [17]. Discrepancies during the data extraction process and critical appraisal were identified and resolved through consensus.

## Results

**Fig. 1:**
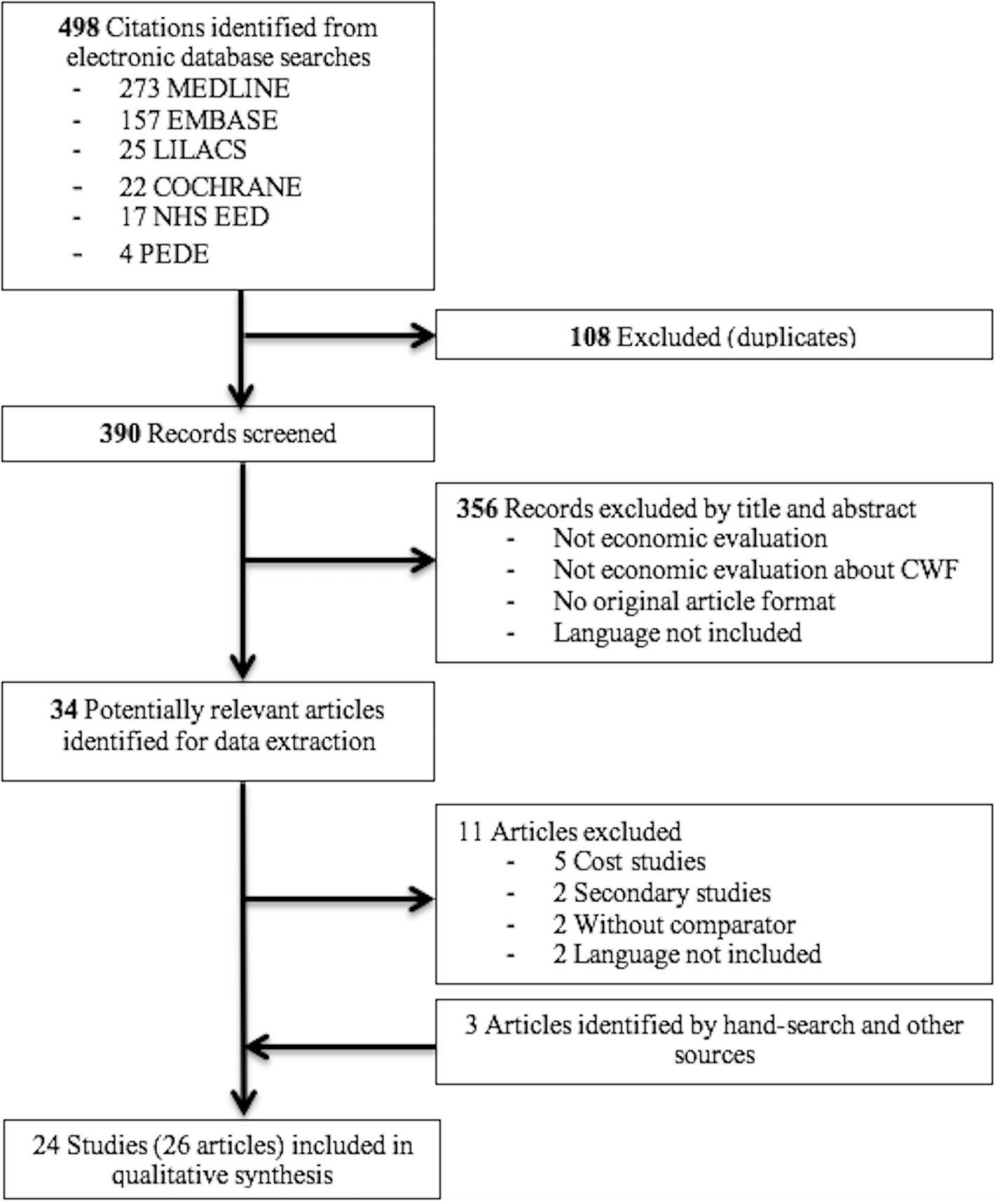
Flowchart presenting the article selection process

A total of 498 articles were found during the database searches. Of these, 108 were duplicates and were eliminated, leaving 390 articles for review. After title and abstract review, 356 articles were excluded. Thirty-four articles were selected for full text review. Of these, five were excluded due to being cost studies [18–22]; two due to being review articles [23, 24]; two did not have comparators [25, 26] and two articles were in French [27, 28]. In addition, three articles were identified by hand search and other online sources. Finally, 26 articles corresponding to 24 studies [29–52] were included, because 2 studies were reported in more than one article [53, 54]. Figure 1 shows the selection process followed.

The main characteristics of the included studies are presented in Table 2 (ordered by year of publication). The studies included were published between 1973 and 2017. By year of publication, by 10-year categories, there is a trend towards a gradual increase in the number of publications; in particular, between 2010 and 2017 (*n* = 11). Eleven studies were cost-benefit analyses; nine were cost-effectiveness analyses; and four were cost-utility studies. Studies were conducted in eight countries: Australia (*n* = 6); USA (*n* = 6); New Zealand (*n* = 4); United Kingdom (*n* = 2); Chile (*n* = 2); Spain (*n* = 2); South Africa (*n* = 1) and Canada (*n* = 1) (See Table 2). Few studies reported the prevalence of caries in the intervening communities, (35 to 96%).

**Table 2:** Characteristics of included studies by publication date

N	Reference	Country	Type of EE	Perspective	Source of effectiveness data	Outcome measure	Time Horizon /Discount Rate	Price Year/ Currency unit	Main Conclusion
1	Davies 1973 [34]	New Zealand	CBA	Public payer	Model (Observational data)	Saving cost dental treatment	10 years/ NR	1965/ NZD	CWF was cost-effective
2	Nelson 1976 [42]	USA	CBA	Payer	Model (Observational data)	DMFS averted	20 years/ 10%	1975/USD	CWF was cost-effective and socially profitable
3	Carr 1980 [49]	Australia	CBA	Public Payer	Model (Observational data)	DMFT averted	10 years/7%	1971/AUD	After seven to 11 years of fluoridation, treatment-cost savings would exceed costs of fluoridation.
4	Doessel 1985 [35]	Australia	CBA	Societal	Model (Observational data)	Saving dental service	15 years/10%	1965/AUD	The study indicates significant economic benefit and CWF was cost-effective.
5	Birch 1986 [30]	United Kingdom	CUA	Payer	Model (Observational data)	QATY	Lifetime/5%	NR/GBP	The lifetime benefit and cost of fluoridation was 18 QATY and GBP1.89 respectively. Cost for QATY produced by water fluoridation was 10.83 pence per QATY
6	Manau 1987 [38]	Spain	CEA	Payer	Model (Observational data)	DMFS averted	20 years/None	1986/ PTS	The CWF was the most cost-effective strategy when compared with other community programs like fluoride mouthrinses or supervised toothbrushing
7	Birch 1990 [29]	United Kingdom	CEA	Payer	Model (Observational data)	dmft/DMFT averted	14 years/5%	1988/GBP	CWF was cost-effective in population with low to high prevalence of caries
8	Millán 1991 [40]	Spain	CBA	Payer	Model (Review)	dmfs averted	20 years/ 6.54%	1988/PTS	The program for the fluoridation of the public water supply in Málaga was profitable from the first year.
9	Murgueytio 1995 [41]	Chile	CBA	Payer	Cohort	Caries averted	10 years/ NR	1995/CLP	CWF was highly cost-effective.
10	Arjunan 2000 [50]	Australia	CBA	Payer	Model (Review)	DMF averted	20 years/ 5%	NR/AUD	Fluoridation of the water supply in small remote communities with a population of more than 1000 is an economically viable investment.
11	Griffin 2001 [36]	USA	CBA	Societal	Model (Review)	Cost averted caries	15 years/ 4%	1995/ USD	Fluoridation was still cost saving for communities of any size if we allowed increment, effectiveness, or the discount rate to take on their worst-case values, individually. For simultaneous variation of variables, fluoridation was cost saving for all but very small communities.
12	Wright 2001 [46]	New Zealand	CBA	Societal	Model (Observational data)	Averted costs of treating caries	30 years/ 5% cost and benefit	1999/ NZD	Fluoridation was cost-saving (dental cost savings exceeded fluoridation costs) for communities above about a thousand people. The true break-even community size may be lower. For smaller communities, fluoridation may be considered cost-effective depending on the non-monetised value assigned to an averted decayed surface.
13	O’Connell 2005 [44]	USA	CBA	Societal	Model	Caries averted	Lifetime/ 3% cost and effect	2003/USD	CWF in Colorado was cost saving. Using lower rates of fluoride effectiveness for areas with fluoride levels greater than 0.3 ppm, CWF remains profitable.
14	Campain 2010 [31]	Australia	CBA	Societal	Model (Synthetic cohorts)	DMFS averted	Lifetime/ 7%/	2005/ AUD	Despite declining levels of dental decay, CWF continues to be a cost-effective preventive measure. However, the cost-effectiveness of CWF was shown to decline with age due to plateauing in decay increment and estimates of higher periodontal treatment needs.
15	Ciketic 2010 [32]	Australia	CUA	Societal	Model	DALY averted	15 years/ 3%	2002/ AUD	Fluoridation remains still a very cost-effective measure for reducing dental decay. CWF was a dominant strategy as more DALYs were saved along with significant cost savings.
16	Cobiac 2012 [33]	Australia	CUA	Payer	Model (Review)	DALY averted	15 years/ 3%	2003/ AUD	Extending coverage of fluoridation to all communities of at least 1000 people will lead to improved population health, with a dominant cost-effectiveness ratio and 100% probability of cost-savings. Extending coverage to smaller communities is not cost-effective.
17	Kroon 2012 [37, 53]	South Africa	CEA/ CBA	Payer	Review	dmft averted Average fee for two surface amalgam	NR	2011/USD	Water fluoridation leads to significant cost savings and remains a cost-effective measure for reducing dental caries, even when the caries-preventive effectiveness is modest.
18	Mariño 2012 [39, 54]	Chile	CEA	Societal	Model (Review)	Caries averted	6 years/3% for cost	2009/CLP	Based on cost required to prevent one carious tooth among schoolchildren, salt fluoridation and CWF were more cost-effective than school-based programmes such as milk-fluoridation, fluoridated mouthrinses, APF-Gel, and supervised toothbrushing with fluoride toothpaste
19	Tchouaket 2013 [45]	Canada	CBA	Societal	Model (Review)	Averted costs of treating caries	20 years/ 3%	2010/CAD	The analyses showed that the water fluoridation program was cost-effective even with a conservatively estimated 1% reduction in dental caries.
20	Edelstein 2015 [51]	USA	CEA	NR	Model (Observational data)	Caries averted	10 years/NR	NR/USD	CWF was the intervention with lowest unit cost and more disease reduction, reaching all children receiving Medicaid regardless of their caries risk
21	Fyfe 2015 [47]	New Zealand	CEA	Societal	Model (Observational data)	DMFT averted	15 years/3,55	2012/NZD	CWF was profitable for communities of more than 5000. For communities of less than 5000, profitability would depend more on the risk profile of the community population.
22	Atkins 2016 [52]	USA	CEA	Payer	Model (Observational data)	Caries averted Full mouth dental reconstructions averted	10 years/3% cost and benefit	2011/USD	While all interventions (CWF, dental sealants, fluoride varnish, tooth brushing with fluoride toothpaste, and conducting initial dental exams on children < 18 months of age) generated a cost saving, CWF had the greatest cost benefit of preventing dental caries.
23	O’Connell 2016 [43]	USA	CBA	Societal	Model	Caries averted	Lifetime/ 3% cost and effect	2013/USD	CWF was cost-effective. The program savings are likely to exceed costs.
24	Moore 2017 [48]	New Zeeland	CUA	Societal	Model (Observational data)	QALYs gained	20 years/3.5%	NZD	Community water fluoridation was highly cost-effective for all but very small communities (< 500).

*CBA* Cost-benefit, *CEA* Cost-effectiveness, *CUA* cost-utility; *CWF* Community water fluoridation, *DALY* Disability-Adjusted Life Year, *EE* Economic evaluation, *QALY* Quality-Adjusted Life Years, *QATY* Quality-Adjusted Tooth Years, *DMFT/S* Decayed, Missing and Filled tooth/tooth surface, *NR* Not reported

The most commonly used perspective was the payor’s (*n* = 12) while eleven studies used the societal one. The perspective is the point of view from which the economic evaluation is carried out and this determines what costs and benefits should be included in the analysis. The payer’s perspective includes only the costs that are directly related to the production of the health service or program whereas the societal perspective also includes the costs incurred by the patient and his/her family, either to access the service or other expenses that can be assumed as a result of the intervention or due to the loss of the productivity [11].

The main comparator was communities without CWF. Only two studies compared the cost-effectiveness of CWF with other preventive measures such as dental sealants, fluoride varnish and, tooth brushing with fluoride toothpaste [52, 54]. With one exception, most of the studies considered a general population older than 5 years of age. The exception was Edelstein and collaborators who included preschoolers [51]. Most of the studies (*n* = 15) adopted time horizons of 15 years or more. However, four studies [30, 43, 44] adopted a lifetime time horizon Regarding the source of data and modelling, most studies (*n* = 21) built Markov models with literature data from observational studies and only one study analyzed data from a cohort study. No studies reported a decision analysis model. (See Table 2). A model is a simple representation of a reality and allows extrapolating of cost and effectiveness parameters beyond the data observed in a clinical trial. It also allows extrapolating of results obtained in one clinical setting in to the general population [55].

Regarding costs and outcomes, all studies incorporated the intervention costs, composed of a one-time investment cost and amortized to obtain annual value; the recurrent fixed costs (costs of maintenance, operation and monitoring); and a variable recurrent cost (chemical and supplies cost) [45]. Additionally, studies from a social perspective included costs for lost productivity due to the time spent in dental care and transportation costs to and from the health center.

Most of the studies reported a caries reduction effects between 25 and 40%. The outcomes most frequently measured were caries averted (*n* = 14) and cost saving of dental treatment (*n* = 4). Cost-utility studies used Disability-Adjusted Life Year (DALY) in two studies, Quality-Adjusted Tooth Years (QATY) in one study, and Quality-Adjusted Life Years (QALY) in another (See Table 2).

All studies supported the use of CWF as a cost-saving strategy when it was compared with a non-fluoridated community, independent of the perspective, time horizon and discount rate applied. Studies also showed that water fluoridation was cost-effective even when the estimated reduction of prevalence was lower than 25% [33, 37, 45]. Moreover, evidence of communities with different concentrations of fluoride in their water supply, found that this measure proved to be cost-effective even in communities with fluoride levels of slightly more than 0.3 ppm [44]. However, two studies showed that in small communities (population of less than 1000 population) the water fluoridation did not achieve cost saving [36, 50]. Additionally, Campain and her collaborators [39], using a time-age model, factored the impact of dental and periodontal treatment needs on the cost savings from community water-fluoridation. They suggested that consideration needed to be given to preventive strategies that contained costs of treatment needs arising out of greater tooth retention in an ageing population (e.g., treatment of periodontal disease). Only one study assessed the cost-effectiveness in preschool populations with different caries risk, and concluded that CWF produced cost savings regardless of caries risk [51].

Most of the studies included showed a moderate methodological quality (12/24) and eight studies met at least 80% of the criteria in the JBI Checklist for Economic Evaluations and were considered high quality (Table 3). All of the studies with fair quality were published before 2000, with the exception of the study by Edelstein and collaborators, published in 2015. The main methodological shortcoming observed was that studies did not provide either a comprehensive description of the alternatives (9/24), did not measure costs and outcomes accurately, or that the sources of information used were unreliable (10/24). Although most of the studies reported a cost-effectiveness ratio for WF, few reported the incremental cost-effectiveness ratio (ICER) (19/24). The ICER informs how much more we must pay to obtain an additional effectiveness unit of the new intervention in relation to its comparator [11]. Furthermore, fifteen studies conducted a sensitivity analysis to investigate uncertainty in estimates of costs or outcomes. Finally, only 10 studies described the study setting adequately and discussed the issues of transferability of findings and how the results were generalizable to other settings with similar characteristics.

**Table 3:** Assessment of methodological quality of included studies

Reference	1. Is there a well defined question?	2. Is there a comprehensive description of alternatives?	3. Are all important and relevant costs and outcomes for each alternative identified?	4. Has clinical effectiveness been established?	5. Are costs and outcomes measured accurately?	6. Are costs and outcomes valued credibly?	7. Are costs and outcomes adjusted for differential timing?	8. Is there an incremental analysis of costs and consequences?	9. Were sensitivity analyses conducted to investigate uncertainty in estimates of cost or consequences?	10. Do study results include all issues of concern to users?	11. Are the results generalizable to the setting of interest in the review?	Quality Rating
Davies 1973 [34]	Unclear	Yes	Yes	Yes	Yes	Yes	No	No	No	Yes	No	Moderate
Nelson 1976 [42]	Yes	Yes	Yes	Yes	No	No	Yes	No	No	Yes	No	Moderate
Carr 1980 [49]	No	Unclear	Yes	Yes	No	Yes	Yes	No	Yes	Yes	No	Moderate
Doessel 1985 [35]	Yes	No	Yes	No	No	No	Yes	No	Yes	Yes	No	Moderate
Birch 1986 [30]	Yes	No	Yes	Yes	Yes	Yes	Yes	No	No	Yes	No	Moderate
Manau 1987 [38]	Yes	No	No	Yes	No	No	No	No	No	Yes	No	Fair
Birch 1990 [29]	Yes	No	No	Yes	No	No	Yes	Yes	Yes	No	No	Moderate
Millán 1991 [40]	Yes	No	No	No	No	No	Yes	Yes	No	No	No	Fair
Murgueytio 1995 [41]	Yes	Yes	Yes	Yes	No	Yes	No	No	No	Yes	No	Moderate
Arjunan 2000 [50]	Yes	Yes	Unclear	Yes	No	Yes	Yes	No	Yes	No	No	Moderate
Griffin 2001 [36]	Yes	Yes	Yes	Yes	No	No	Yes	Yes	Yes	Yes	No	Moderate
Wright 2001 [46]	Yes	No	Yes	Yes	Yes	No	Yes	No	Yes	Yes	Yes	Moderate
O’Connell 2005 [44]	Yes	Yes	No	Yes	Yes	No	Yes	No	Yes	Yes	Yes	Moderate
Campain 2010 [31]	Yes	Yes	Yes	Yes	Yes	Yes	Yes	No	Yes	Yes	Yes	Good
Ciketic 2010 [32]	Yes	Yes	No	No	Yes	No	Yes	No	Yes	Yes	No	Moderate
Cobiac 2012 [33]	Yes	Yes	Yes	Yes	Yes	Yes	Yes	No	Yes	Yes	Yes	Good
Kroon 2012 [37, 53]	Yes	Yes	No	Yes	Yes	Yes	No	No	No	No	No	Moderate
Mariño 2012 [39, 54]	Yes	No	Yes	Yes	No	Yes	Yes	Yes	Yes	Yes	Yes	Good
Tchouaket 2013 [45]	Yes	Yes	Yes	Yes	Yes	Yes	Yes	No	Yes	Yes	Yes	Good
Edelstein 2015 [51]	Yes	Yes	No	Yes	No	No	No	No	No	No	No	Fair
Fyfe 2015 [47]	Yes	Yes	Yes	Yes	Yes	Yes	Yes	No	Yes	Yes	Yes	Good
Atkins 2016 [52]	Yes	Yes	Yes	Yes	Yes	Yes	Yes	Yes	No	Yes	Yes	Good
O’Connell 2016 [43]	Yes	Yes	Yes	Yes	Yes	Yes	Yes	No	Yes	Yes	Yes	Good
Moore 2017 [48]	Yes	No	Yes	Yes	Yes	Yes	Yes	No	Yes	Yes	Yes	Good

## Discussion

A scoping review of studies concerning EE of CWF for dental caries prevention, published after 1973, revealed 24 EEs of CWF. Most of the studies identified were either cost effectiveness or cost benefit analyses. The majority of studies, even when they reported cost per carious lesion averted, presented final analyses in terms of monetary costs and benefits. Overall, findings indicated that there were significant economic benefits to using CWF, representing an efficient use of financial resources. Notably, this general conclusion remains when using different methods of evaluation or different settings.

Nonetheless, few studies addressed variables such as the size of the community, the declining dental caries incidence and greater tooth retention; or the ageing of the population. These factors would impact on the expected benefits and costs when modelling future outcomes. Griffin and her collaborators’ [36] also addressed the question of whether, in the face of declining dental caries incidence, CWF is still economically and socially justifiable CWF. Regarding the extent to which net economic benefits exist in small communities, studies indicated a population of 1000 population as the cut-off point for cost-effectiveness [33, 50]. Moore and collaborators., meanwhile, showed that CWF was highly cost-effective for all but very small communities (< 500) [48]. The main justification provided for these studies was that the cost of its implementation and maintenance is greater than the averted costs, despite the higher rates of caries in more regional and remote populations. These results should be considered with caution, since the studies included, did not always consider the costs of complex treatments (root canal treatment, implants, crowns, etc.), assuming that the costs of treating adverse effects were negligible, or that the effectiveness was the same regardless of age. However, two studies questioned that conclusion and indicated that CWF in communities of any size would be cost saving [36, 46].

As mentioned, most of the included studies considered that the adverse effects of CWF, such as dental fluorosis, were negligible, and failed to include the costs associated with the treatment of these conditions. Although, the problems caused in the less severe forms of fluorosis are even considered to be aesthetically pleasing [56], the evidence shows that fluorosis causes aesthetic problems to up to 40% of those surveyed, when considering fluorosis of any level [8]. The treatment of fluorosis ranges from bleaching, in the case of mild fluorosis, to complex treatments such as veneers and crowns in the case of moderate or severe fluorosis [57]. When the time horizon of a lifetime is used, the costs associated with the treatment and retreatment of dental fluorosis can be significant and should therefore be included.

Furthermore, it must be noted that programs and interventions in health might be cost-inefficient, but could be used regardless because of ethical or cultural considerations. For example, life-saving interventions, or those that improve quality of life.

Campain and collaborators [39] took into consideration the effects of an ageing population, lower rates of edentulism, and consequently higher rates of treatment need for the gums and supporting tissues of the teeth. They found that CWF continued to be a cost-effective preventive measure. However, the study suggested that CWF might stop being cost-effective due to the additional costs of treatment of periodontal disease and replacement/retreatment of old restorations, etc., highlighting the need to incorporate preventive measures in oral health.

Only four cost-utility studies were identified. CUA was developed to help decision-makers compare the value of alternative interventions that have different health outcomes. In those cases, CUA facilitates these comparisons without the need to place monetary value on different health states, being more useful when alternative treatment strategies produce different types of outcomes [58]. Since CUA reflects the preference that the patient’s preference has regarding a state of health, information related to the patient is lost at the time of making decisions. When using another outcome measure. For example, patients can choose a tooth-colored composite resin instead of an amalgam for restoration, despite the costs involved. The absence of CUA may be due to the absence of suitable instruments to measure utility in oral health [59]. On the other hand, evidence shows that CUAs tend to be less favorable than CEAs in short time horizons, which may discourage their use [60].

Studies included in this review were conducted in eight countries, with half of the studies based either in Australia or in the USA. EE are very context specific, and most likely reflect the local conditions from those countries only [61]. Thus, care must be exerted when generalizing results to another jurisdiction, as it remains difficult to compare economic results from one country to another [61] or even within a jurisdiction from a given year to another, and the cost of a technology may also vary after its initial introduction [62]. There are many reasons for the difficulty of comparing costs, among them: differences in price of resources; variability in willingness to pay for health and health care; variations in prices of health consequences; variation in approaches to treatment and resource use [63]. Furthermore, the risk behaviors of the population, health care infrastructure, and a society’s ideological and ethical norms could also differ [64].

Therefore, the methodological quality of the existing EEs plays a fundamental role when transferring an economic evaluation to another context [65] and those who conduct EE should pay attention to methodological issues. In the present review, most studies used modelling to asses cost, benefits and effectiveness. A crucial feature of models is their transparency and reproducibility in order to allow adaptation to different scenarios [55]. The effectiveness of any intervention must be based on the best available data and clearly stated. Therefore, policy makers should be able to identify if the model reflects the usual practice and whether they used an adequate comparator. They must also identify whether the information on which the model is based is the best quality available according to the technology evaluated, population studied and study perspective. A number of the EE included in this review failed some of these aspects.

Differences in caries rates between fluoridated and non-fluoridated communities reported by the included studies are in the order of 30%. Although other preventive strategies such as fluoride varnishes, dental sealants or brushing with fluoride toothpaste can be more effective and also costs saving, drinking water has the advantage of reaching people of all ages, and education and income levels within a community [66]. Manau and collaborators showed that CWF was the most cost-effective strategy when compared with other community programs such as fluoride mouthrinses or supervised toothbrushing [38]. This is reinforced by Mariño and his collaborators [54] who found that CWF was more cost-effective than school-based programmes such as milk-fluoridation, fluoridated mouthrinses or APF-Gel.

The time period of an analysis is usually short and not related to the lifespan of the population. Because of the nature of analyses required to inform fluoridation decisions, prospective epidemiological data for a lifetime, or at least from early childhood to advanced old age, are needed. Only four studies [30, 43, 44] used a time horizon of lifetime, although the methodological guidelines for the preparation of EE recommend a lifetime horizon for chronic diseases such as tooth decay [67].

Although we were systematic in our review, it is possible that we may have missed publications. However, we believe that this was minimized due to the sensitive search strategy used, the additional search of references by hand and the double independent review process used. Additionally, it was not possible in this review to conduct a full validation of each model.

## Conclusion

A scoping review was conducted to provide a review of the evidence concerning EE of CWF for dental caries prevention. Findings indicated that CWF represents an appropriate use of communities’ resources, using a range of economic evaluation methods and in different locations. In accordance with the evidence found, future EEs of CWF should include a broad perspective that covers a lifetime temporal horizon and includes not only the direct cost of investment and operation, but also other negative and positive intangible costs of water fluoridation, such as, political cost (e.g., cost promotion of CWF) and adverse effect cost (e.g., treatment of fluorosis, which might include complex rehabilitation), among others. In addition, future studies should consider the decline in caries rates over time. Furthermore, the outcome measure should also consider changes in quality of life, subsequent to changes in oral health status.

Additionally, in view of changing technology, disease prevalence and socio demographic profile of the population, evaluation and research on the cost-effectiveness of CWF should be ongoing and locally based. Significantly, there are not conclusive results in communities less than 1000, or in temporal horizons lower than 10 years. Finally, to facilitate the interpretation of the results by decision makers in health, the results should be summarized through the incremental cost-effectiveness ratio (ICER).

## Supplementary information

**Additional file 1.** Search strategy used in each database.

## Data Availability

The datasets used and/or analyzed during the current study are available from the corresponding author upon reasonable request.

## Abbreviations

CBA: Cost-benefit
CEA: Cost-effectiveness
CUA: cost-utility
CWF: Community water fluoridation
DALY: Disability-Adjusted Life Year
EE: Economic evaluation
QALY: Quality-Adjusted Life Years
QATY: Quality-Adjusted Tooth Years
