# Supplementary material for: Economic evaluations in water-fluoridation: a scoping review
Source: BMC Oral Health. 2020 Apr 16;20:115. doi: 10.1186/s12903-020-01100-y (PMC7164347; doi:10.1186/s12903-020-01100-y)
Supplement: Supplementary file 1 — Additional file 1. Search strategy used in each database. [file 12903_2020_1100_MOESM1_ESM.docx]

**Supplementary material: Search Strategy Used in Each Database**

| **Source** | **Strategy** |
| --- | --- |
| Medline | ((((((((((((("Fluorides"[Mesh]) OR "Fluoridation"[Mesh]) OR "Fluorine"[Mesh]) OR "fluorene" [Supplementary Concept]) OR Fluorid*) OR fluorin*))))))) AND (((((("Drinking Water"[Mesh]) OR "Water Supply"[Mesh]) OR "Water"[Mesh]) OR water)))) AND (((((((("Costs and Cost Analysis"[Mesh])) OR "Cost-Benefit Analysis"[Mesh]) OR cost-benefit) OR cost-utility) OR cost-effectiveness) OR economic analysis) OR economic evaluation) |
| Embase | #18. #5 AND #10 AND #17  #17. #11 OR #12 OR #13 OR #14 OR #15 OR #16  #16. fluorin*  #15. fluorid*  #14. 'fluorene'/exp  #13. 'fluorine'/exp  #12. 'fluoridation'/exp  #11. 'fluoride'/exp  #10. #6 OR #7 OR #8 OR #9  #9. water  #8. 'water supply'/exp  #7. 'drinking water'/exp  #6. 'water'/exp  #5. #1 OR #2 OR #3 OR #4  #4. cost AND benefit:ti,ab  #3. cost AND utility:ti,ab  #2. cost AND effectiveness:ti,ab  #1. 'cost benefit analysis'/exp |
| NHS EED | 1 MeSH DESCRIPTOR Cost-Benefit Analysis EXPLODE ALL TREES  2 (cost-effectiveness)  4 (cost-benefit)  5 (cost-utility)  6 #1 OR #2 OR #4 OR #5  7 MeSH DESCRIPTOR Drinking Water EXPLODE ALL TREES  8 MeSH DESCRIPTOR Water Supply EXPLODE ALL TREES  9 (water)  10 #7 OR #8 OR #9  11 MeSH DESCRIPTOR Fluorides EXPLODE ALL TREES  12 MeSH DESCRIPTOR Fluoridation EXPLODE ALL TREES  13 MeSH DESCRIPTOR Fluorine EXPLODE ALL TREES  14 (fluorid*)  15 (fluorin*)  16 #11 OR #12 OR #13 OR #14 OR #15  17 #6 AND #10 AND #16 |
| Lilacs | (MH:"Costs and Cost Analysis" OR MH:"Cost-Benefit Analysis" OR cost-effectiveness OR cost-utility OR cost-benefit) AND (MH:"Drinking Water" OR MH:"Water Supply" OR Water) AND (MH:"Fluoridation" OR MH:"Fluorides" OR MH:"Fluorine" OR fluorid$ OR fluorin$) |
| Cochrane Central | #1 MeSH descriptor: [Cost-Benefit Analysis] explode all trees  #2 cost-benefit  #3 cost-effectiveness  #4 cost-utility  #5 #1 OR #2 OR #3 OR #4  #6 MeSH descriptor: [Water] explode all trees  #7 MeSH descriptor: [Water Supply] explode all trees  #8 MeSH descriptor: [Drinking Water] explode all trees  #9 water  #10 #6 OR #7 OR #8 OR #9  #11 MeSH descriptor: [Fluorides] explode all trees  #12 MeSH descriptor: [Fluoridation] explode all trees  #13 MeSH descriptor: [Fluorine] explode all trees  #14 fluorid*  #15 fluorin*  #16 #11 OR #12 OR #13 OR #14 OR #15  #17 #5 AND #10 AND #16 |
| PEDE | (TITLE_ABSTRACT_KEYWORDS "water(-\| )fluoridation") |
